# Women participating in a web-based preconception study have a high prevalence of risk factors for adverse pregnancy outcomes

**DOI:** 10.1186/1471-2393-14-169

**Published:** 2014-05-17

**Authors:** Elisabetta Pandolfi, Eleonora Agricola, Micaela Veronica Gonfiantini, Francesco Gesualdo, Mariateresa Romano, Emanuela Carloni, Pierpaolo Mastroiacovo, Alberto E Tozzi

**Affiliations:** 1 Bambino Gesù Children’s Hospital IRCCS, Epidemiology Unit, Piazza S. Onofrio 4, 00165 Rome, Italy; 2 Departement of Onco-Ematology and Transplantation Medicine, Bambino Gesù Children’s Hospital IRCCS, Piazza S. Onofrio 4, 00165 Rome, Italy; 3 Alessandra Lisi International Centre on Birth Defects and Prematurity, Via Carlo Mirabello 14, 00192 Rome, Italy

**Keywords:** Adverse pregnancy outcomes, Prevalence, Preconception care, Maternal-child health services

## Abstract

**Background:**

Adverse pregnancy outcomes (APOs) can be increased by preconception risk factors and lifestyles.

We measured the prevalence of preconception risk factors for APOs in a population of Italian women of childbearing age enrolled in a web-based study.

**Methods:**

Participants were enrolled through a web platform (http://www.mammainforma.it). After enrollment, participants filled in a questionnaire regarding socio-demographic characteristics, clinical data and preconception risk factors for adverse pregnancy outcomes. Through logistic regression, we explored how the prevalence of risk factors was affected by age, education level, employment, parity, physician’s recommendation and knowledge of the specific risk factor.

**Results:**

We enrolled a total of 728 women. Sixty-two percent had a University degree, 84% were employed and 77% were planning their first pregnancy.

Nearly 70% drank alcohol in any quantity; 16% were smokers; 6% was underweight; 21.4% was overweight; 51.6% did not assume folic acid; 22% was susceptible to rubella, 44.5% to hepatitis b and 13.2% to varicella.

According to the multivariate analysis, compared to women who already had at least one pregnancy, nulliparous women had a higher BMI [OR 1.60 (CI 1.02;2.48)] and were less likely to be susceptible to rubella [OR 0.33 (CI 0.20;0.58)] and to be consuming alcohol [OR 0.47 (CI 0.31;0.70)] or cigarettes [OR 0.48 (CI 0.26;0.90)].

Appropriate knowledge was associated with a correct behavior regarding smoking, drinking alcohol and folic acid supplementation.

**Conclusions:**

This study shows that the prevalence of risk factors for APOs in our population is high.

Interventions aimed at reducing risk factors for APOs are needed and, to this purpose, a web intervention may represent a feasible tool to integrate tailored information and to inform preconception counseling targeting a specific group of women planning a pregnancy who are engaged on the web.

## Background

Adverse pregnancy outcomes (APOs) are defined as events that reduce the chance of having a healthy newborn. This definition includes spontaneous abortion, preterm delivery, restricted fetal growth, birth defects. Not only during pregnancy, but also during the preconception period, a number of factors may increase the risk of APOs: smoking [1], excessive alcohol intake, being overweight or underweight and being affected by a chronic disease (eg. diabetes) [2-8]. Therefore, the period before a child is conceived is crucial for the prevention of APOs. It is well established that consumption of folic acid before conception and during the early weeks of pregnancy significantly decreases the risk of neural tube defects (NTDs) [9]. Moreover, during the preconception period, assessment of immunization status and consequent vaccinations may prevent miscarriage, fetal death, or birth defects caused by vaccine-preventable infectious diseases contracted during the first 16 weeks of pregnancy [10].

An interesting target population for preconception preventative interventions is represented by women of childbearing age who are engaged on the web: a large proportion of this group of women frequently search for health information online [11]. In a recent survey, we showed that web-engaged women planning a pregnancy search for information on Google using preconception-related and pregnancy-related keywords [12]. This population is probably characterized by a peculiar profile and by specific preconception risk factors. A characterization of this profile can inform tailored, possibly cheap web-based prevention interventions.

In the context of a web-based study aimed at informing women of childbearing age on preconception risk factors for APOs, we investigated the prevalence of such risk factors in the population of women participating in the study.

## Methods

### Study design and population

We conducted a web-based study from September 2011 to May 2013 on a population of women of childbearing age planning a pregnancy within the following year. The project had research purposes, and was promoted by the Bambino Gesù Children’s Hospital, a comprehensive medical centre for pediatric healthcare and research. Participants were enrolled through a web platform dedicated to women planning a pregnancy (https://www.mammainforma.it), where they responded to a set of questions and were subsequently provided with a document, tailored to their profile, with recommendations for prevention.

Eligibility and exclusion criteria were automatically reviewed on the web platform. The eligibility criteria for enrollment were: 1) female gender; 2) age 18–45 years; 3) residence in Italy; 4) Italian language spoken; 5) plan of pregnancy within the next year; 6) active email address and Internet access; 7) web informed consent signed. Women with an ongoing pregnancy were excluded from the study. When eligibility criteria were not met, the web system automatically blocked-out the enrollment generating a specific warning message. Consent to participation in the study was obtained through an online form. Participants were never interviewed in person.

After enrollment, participants were asked to fill-in a questionnaire collecting social and demographic data (age, education level, region of residence, parity, employment), personal medical history (gynecologist or obstetrician visit in the past 12 months, Pap smear test in the last five years), risk factors for APOs and knowledge of risk factors for APOs (Additional file 1).

Specifically, the following risk factors were studied: being overweight or obese (BMI ≥ 25), being underweight (<18.5); smoking; alcohol consumption; no folic acid intake; family history of malformations; consanguinity; diagnosis of maternal underlying diseases (including hypertension, hyperphenylalaninemia, phenylketonuria, hypothyroidism, hyperthyroidism, epilepsy, diabetes type 1, asthma); susceptibility to vaccine preventable diseases; currently used medications.

Once the questionnaire was fully completed and confirmed, the platform generated a downloadable document that included a set of recommendations tailored on the participant’s profile, based on the answers to the questionnaire. The document suggested interventions to eliminate or reduce the risk factors and invited the participants to have a preconception counseling. The recommendation set was based on the guidelines of the American College of Obstetricians and Gynecologists [13].

We promoted the project website through Facebook posts published twice a week for 2 months and through articles freely published on 10 web sites dedicated to women’s health and family care. No paid advertisement was ever used and no incentives were offered for participation in the study.

### Ethical approval

The study was approved by the Bambino Gesù Children’s Hospital’s ethical committee.

### Definitions and statistical analysis

We described sociodemographic variables, medical history, and prevalence and knowledge for each item (as indicated in the previous section) as mean and standard deviation (SD) or proportions and 95% confidence intervals (CI), as appropriate. Susceptibility to vaccine preventable diseases was defined as: a) a negative serological test; b) no test ever performed and no specific immunization received. Women who had a positive serological test, or had received a specific immunization, or, for varicella only, had recall of the clinical disease, were categorized as “protected”.

Through logistic regression, we explored how the prevalence of risk factors was affected by age, education level, employment, parity, physician’s recommendation and knowledge of the specific risk factor. Risk factors studied in the multivariate analysis were: being underweight, overweight or obese, susceptibility to rubella, varicella and hepatitis b, no folic acid intake, smoking (any quantity) and drinking alcohol (any quantity).

We used the STATA 12 statistical package to perform the statistical analysis.

## Results

We enrolled a total of 728 women. Socio-demographic characteristics are described in Table 1. Mean age was 32.6 years. The majority of participants lived in the northern regions of Italy, had a university degree, were employed and were planning their first pregnancy. Most participants had attended a visit with a gynecologist-obstetrician less than a year before ( and had performed a Pap smear less than 5 years before.

**Table 1:** Characteristics of participants (N = 728)

	**Women included in the survey**
**Age, mean (SD)**	32.60 (4.7)
**University Degree, N (%)**	449 (61.7)
**Employed, N (%)**	616 (84.6)
**Residence**	
North, N (%)	384 (52,7)
Center, N (%)	213 (29,2)
South, N (%)	131 (17.9)
**Planning first pregnancy, N (%)**	559 (76.9)
**Ob-Gyn visit in the last year, N (%)**	607 (83.5)
**Pap-test in the last 5 years N (%)**	662 (91.1)

Prevalence of risk factors for APOs is described in Table 2. A proportion of 14.6% [95% CI (.121;.173)] of women were affected by an underlying disease.

**Table 2:** Prevalence of risk factors for APOs in the study population

	**Risk factors prevalence, n (%)**	**95% CI**
**BMI < 18.5**	46 (6.3)	(.047;.083)
**BMI ≥ 25**	156 (21.4)	(.185;.245)
**Underlying diseases**	106 (14.6)	(.121;.173)
**Current drug assumption**	144 (19.8)	(.169;.229)
**Consanguinity**	21 (2.9)	(.018;.044)
**Family history of malformation**	90 (12.4)	(.101;.150)
**Need vaccine for rubella**	161 (22.1)	(.191;.253)
**Need vaccine for varicella**	96 (13.2)	(.108;.159)
**Need vaccine for hepatitis B**	324 (44.5)	(.408;.482)
**No folic acid assumption**	375 (51.6)	(.478;.552)
**Smoking, any quantity**	117 (16.1)	(.135;.189)
**Drinking alcohol, any quantity**	501 (68.8)	(.653;.722)

Almost 20% of participants used medicines; nevertheless, only 2.5% used drugs that are classified as dangerous during pregnancy (neuropsychiatric drugs, NSAID).

Nearly 70% [95% CI (.653;.722)] reported to drink any quantity of alcohol; although heavy drinkers (more than two glasses of wine per day) were only 1% in our population, nearly 10% of participants declared to drink at least one glass of wine or beer every day.

Sixteen percent [95% CI (.135;.189)] reported to be smokers; of these, 79% smoked up to 10 cigarettes per day.

Six percent [95% CI (.047;.083)] was underweight, one fifth [21.4% (95% CI .185;.245)] was overweight and more than a half [51.6% (95% CI .478;.552)] did not assume folic acid.

Regarding vaccine preventable diseases, 22% [95% CI (.191;.253)] of participants were susceptible to rubella, 44.5% [95% CI (.408;.482)] to hepatitis b and 13.2% [95% CI (.108;.159)] to varicella.

Table 3 shows participants’ knowledge for each single risk factor.

**Table 3:** Specific knowledge of risk factors for APOs

**Risk factors**	**Specific knowledge of risk factors for APO (%)**	**95% CI**
**BMI < 18.5**	656 (98.5)	(87.8;92.1)
**BMI > =25**	229 (34.4)	(28.2;35)
**No underlying diseases treatment**	498 (74.8)	(65;71.8)
**Current drug assumption**	585 (87.8)	(77.3;83.1)
**Need vaccine for rubella**	542 (81.4)	(71.2;77.5)
**Need vaccine for varicella**	542 (81.4)	(71.2;77.5)
**Need vaccine for hepatitis B**	542 (81.4)	(71.2;77.5)
**No folic acid assumption**	638 (95.8)	(85.1;89.9)
**Smoking, any quantity**	641 (96.2)	(85.5;90.3)
**Drinking alcohol, any quantity**	428 (64.3)	(55.2;62.3)

Knowledge level on appropriate preconception lifestyles and behaviors was overall high in our population. Nearly 96% of women knew that folic acid supplementation in the preconception period has a benefit against APOs. Nearly 65% [95% CI (55.2;62.3)] knew that drinking alcohol in any quantity is harmful and 96.2% [95% CI (85.5;90.3)] recognized smoking as a risk factor.

Regarding knowledge about the risks of being overweight, only 34.4% [95% CI (28.2;35)] of participants were correctly informed.

Nearly 82% [95% CI (71.2;77.5)] of women knew that an assessment of their immunizations status for rubella, varicella and hepatitis b before pregnancy was important.

According to the multivariate analysis (Table 4), compared to women who already had at least one pregnancy, nulliparous women had a higher BMI and were less likely to be susceptible to rubella and to be consuming alcohol or cigarettes.

**Table 4:** Determinants of risk factors for APOs

**Risk factors**	**Ob-Gyn visit in the last year**	**Knowledge of risk factors for APO**	**Planning first pregnancy**
	**OR (95% CI)**	**OR (95% CI)**	**OR (95% CI)**
**BMI < 18.5**	1.17 (0.90;1.06)	1(0.00-/)	1.22 (0.55;2.69)
**BMI ≥ 25**	0.70 (0.42;1.14)	1.04 (0.70;1.55)	**1.60 (1.02;2.48)**
**Need of vaccine for rubella**	**0.6 (0.37;0.97)**	0.75 (0.48;1.19)	**0.33 (0.20;0.58)**
**Need of vaccine for varicella**	1.13 (0.60;2.13)	0.80 (0.46;1.39)	0.73 (0.40;1.31)
**Need of vaccine for hepatitis B**	1.08 (0.70;1.68)	0.73 (0.49;1.10)	0.79 (0.53;1.17)
**No folic acid assumption**	**0.23 (0.14;0.38)**	**0.27 (0.10;0.69)**	1.42 (0.97;2.12)
**Smoking, any quantity**	1.21 (0.93;1.03)	**0.10 (0.04;0.24)**	**0.48 (0.26;0.90)**
**Drinking alcohol, any quantity**	1.1 (0.64;1.60)	**0.70 (0.48;0.99)**	**0.47 (0.31;0.70)**

Footnote: the bold character underlines those determinants significantly associated with APOs.

Appropriate knowledge of specific risk factors was associated with a correct behavior regarding smoking, drinking alcohol and folic acid supplementation.

Finally, women who had visited a gynecologist during the previous year were less likely to be susceptible to rubella and were taking folic acid more often compared to those who had not been recently visited.

## Discussion

Our web-based study shows that Italian women planning a pregnancy report a high prevalence of risk factors for APOs.

In Italy, APOs represent a significant burden. Specifically, birth defects account for almost 2% of live births, fetal deaths and termination of pregnancy for fetal anomaly following prenatal diagnosis. The most common reported anomalies are congenital heart defects (around 0.7%), neural tube defects (0.05%) and oro-facial clefts (0.1%) [14].

Several information campaigns have been conducted in Italy promoting folic acid supplementation before pregnancy [15,16]. Nevertheless, more than a half of participants declared that they were not taking folic acid supplementation. This is an alarming result, taking into account current evidence showing the very high efficacy of folic acid supplementation for neural tube defect prevention [17]. The role of health care providers is crucial regarding prevention of neural tube defects through folic acid supplementation: according to the multivariate analysis, participants with a good knowledge of folic acid benefits and those who had attended a gynecologic visit during the previous year were more frequently on folic acid supplementation. Information campaigns on this subject should be strongly implemented, possibly taking advantage of novel media and marketing strategies directed to this kind of population.

The prevalence of smokers in our population (16%) was only slightly lower than that in the general female population of the same age group, which is estimated between 19 and 22% [18].

Although we do not have information on smoking habits in participants before starting to plan a pregnancy, our results suggest that probably only a small proportion of smokers quit smoking in the preconception period. Nevertheless, appropriate knowledge on smoking in the preconception period as a risk factor for APOs was associated with a lower prevalence of the risk factor. This confirms that more incisive and effective information campaigns are needed.

The proportions of participants drinking alcohol in any quantity (70%) and of those consuming at least one alcohol unit per day (10%) were higher compared to figures from the general female population: as of 2012, 44.3% of Italian women aged 18–44 years drank at least 1 alcohol unit per month; on the other hand, 2% of Italian women of the same age group drank at least one alcohol unit per day [19].

It is likely that the higher figures identified by our study are confounded by the high socio-demographic status of our population. In Italy, in fact, women with a higher socio-demographic status drink alcohol more frequently [19]. Compared to the general population of 15–64 ys age groups, the participants in our study had a significantly higher employment rate (84.6% vs about 50.5%) [20] and a higher education level (61.7% with a university degree vs about 20.7%) [21]. Therefore, a higher proportion of people drinking alcohol was expected. Information campaigns should focus on this segment of highly educated and employed population regarding the risk of alcohol consumption during the preconception period. Moreover, recent data indicate that, in the general population, almost 1 on 3 women with an ongoing pregnancy drink alcohol in any quantity, despite current recommendations, showing that this information is probably neglected by health professionals and public health agencies.

Another neglected area, which deserves implementation, is represented by risks connected with overweight and obesity in the preconception period: overweight was a relatively common risk factor in our sample, and at the same time it was the item with the lowest level of knowledge among participants.

Susceptibility to vaccine preventable diseases was high. In Italy, a national campaign for congenital rubella elimination in adults has been implemented since 2003; varicella immunization is not actively offered throughout the country but is recommended in adolescents with no varicella history; hepatitis B immunization has been universally offered to infants in the first year of life since 1991, and a screening is systematically offered during pregnancy.

Our data show a high susceptibility level in line with Italian seroepidemiological figures and immunization rates that show a high proportion of women of childbearing age susceptible to rubella [22].

These data provide an estimate of women who need medical attention and that should be offered immunization. Participants who had attended a gynecologist visit during the previous year were more frequently protected against rubella.

Primiparous and multiparous woman were found more at-risk regarding smoking, drinking alcohol and rubella susceptibility, compared to women planning their first pregnancy, despite a good knowledge of the respective risk factors for APOs. This result may be explained by a more “relaxed” attitude after the first pregnancy regarding preconception risks, both by women and by physicians.

Based on the multivariate analysis, it seems that preconception counseling during a recent gynecologist visit does not affect the prevalence of a number of risk factors, including underweight, overweight, smoking, alcohol drinking and susceptibility to varicella and hepatitis b. This items should be more thoroughly discussed by health care professionals in contact with women planning a pregnancy.

The main limitation of our study concerns a potential selection bias: we have likely selected a population of highly educated and employed women, with an easier access to the web, who are probably better informed and who usually attend medical screenings when planning a pregnancy. In this light, the high prevalence of risk factors identified by our study is even more alarming. Taking into account that our sample is different from the general population, our results are probably not easily generalizable; nevertheless, our sample is likely representative of the population of women that frequently use the Internet to search for health information, which is a specific group that can be easily reached by targeted, tailored and possibly inexpensive web-based interventions.

A strength of our study is that, while most studies on preconception risks focused on a small number of risk factors [23], we took into account a large number of potential risk factors, which were included in the multivariate analysis, thus allowing to evaluate multiple potential confounders.

## Conclusions

In conclusion, our study shows that interventions aimed at reducing risk factors for APOs are needed and, to this purpose, a web intervention may represent a valid tool to integrate tailored information and to inform preconception counseling targeting a specific population group. A study of this kind may also allow to longitudinally monitor the prevalence of preconception risk factors on this at-risk population, at relatively low costs.

## Abbreviations

APOs: Adverse pregnancy outcomes; BMI: Body Mass Index.

## Competing interests

All authors declare that they have no competing interests.

## Authors’ contributions

EP coordinated the study, designed the study and participated in the writing process and in the data review. EA designed the study, drafted the manuscript and participated in data review. MVG, FG and MR revised the final version of the manuscript. EC performed the statistical analysis. PM conceived the study, participated in its design, AET conceived the study, participated in its design and coordination and drafted the manuscript. All authors read and approved the final manuscript.

## Pre-publication history

The pre-publication history for this paper can be accessed here:

http://www.biomedcentral.com/1471-2393/14/169/prepub

## Supplementary Material

Additional file 1 Questionnaire PROFILE. Click here for file
