# Supplementary material for: Women participating in a web-based preconception study have a high prevalence of risk factors for adverse pregnancy outcomes
Source: BMC Pregnancy Childbirth. 2014 May 17;14:169. doi: 10.1186/1471-2393-14-169 (PMC4038071; doi:10.1186/1471-2393-14-169)
Supplement: Additional file 1 — Questionnaire PROFILE. [file 1471-2393-14-169-S1.doc]

| **Questionnaire PROFILE** | |
| --- | --- |
| **1.General Informations** | |
| 1.1 | How old are you? |
| 1.2 | Where do you live? |
| 1.3 | Education |
| 1.4 | Employment |
| **2.Health Status** | |
| 2.1 | How much do you weight? (kg) |
| 2.2 | How tall are you (cm) |
| 2.3 | Did you have a obstetrician/gynaecologist check up visit in the last year? |
| 2.4 | Did you perform a Pap smear test in the last five years? |
| 2.5 | Do you have: |
|  | a) High blood pressure? |
|  | b)Phenylketonuria or hyperphenylalaninemia? |
|  | c) Hypothyroidism? |
|  | d) Diabetes? |
|  | e) Epilepsy? |
|  | f) Asthma? |
|  | h) Hyperthyroidism? |
|  | e) Are you taking any medicine (including over the counter medicine)? |
|  | If yes, what? |
| **3.Family History** | |
| 3.1 | Are You and your partner blood relatives? |
| 3.2 | Is there any birth defect, disability, chronic or genetic diseases In your or your partner's family ? |
| **4. Infectious diseases** | |
| 4.1 | Do you remember having had chickenpox? |
| 4.2 | Have you ever performed a test to verify whether you had or you have been vaccinated for: |
|  | a) Rubella |
|  | b) Varicella |
|  | f) Hepatitis B |
| 4.3 | Did you get vaccination for: |
|  | a) Rubella |
|  | b) Hepatitis B |
|  | c) Varicella |
| **5. Lifestyle** | |
| 5.1 | Do you smoke? |
|  | - If yes, how many cigarettes per day: |
| 5.2 | Do you drink alcohol even occasionally (any quantities) in small quantities? |
|  | - If yes, how much: |
| 5.3 | Are you taking any multivitamins or folic acid? |
| **6. Previous pregnancies** | |
|  | a) Have you had more than one miscarriage? |
|  | b) Have you ever had any abortion for medical reasons? |
|  | c) Do you have children? |
| **Questionnaire KNOWLEDGE** | |
| 1.1 | Are you informed on the general preconception behavior to increase the chance of having a healthy baby? |
| 1.2 | In your opinion folic acid consumption could: |
| 1.3 | Which of these compounds, reduces the risk that the child will be born with a birth defect? |
| 1.4 | Now that you've decided to have a baby, when do you think you should schedule your first visit to a health professional (GP, gynaecologist)? |
| 1.5 | Are you aware that some malformations or genetic diseases in earlier children or in your family can be inheritable? |
| 1.6 | When does the risk of having a child with Down syndrome increase? |
| 1.7 | How much do you think it is important to begin a pregnancy with a normal weight? |
| 1.8 | Compared to women with a normal weight, overweight or obese women have a higher risk of having the following problems during pregnancy, except for one. Which one? |
| 1.9 | Which of the following diseases or maternal conditions can frequently damage the baby? |
| 1.10 | Are you aware of the damages that smoking can cause in a woman planning a pregnancy? |
| 1.11 | According to your opinion, which of the following statements is true |
| 1.12 | Can Alcohol consumption before pregnancy can cause problem |
| 1.13 | Do you know that it is important to assess your vaccination status before you get pregnant? |
| 1.14 | In your opinion, what vaccination for is important to get when you are not protected? |

| **Answers Questionnaire PROFILE** | | | |
| --- | --- | --- | --- |
| **1.General Informations** | | | |
| 1.1 | Open ended question | | |
| 1.2 | | PIEMONTE | | --- | | VALLE D'AOSTA | | LOMBARDIA | | TRENTINO-ALTO ADIGE | | VENETO | | FRIULI-VENEZIA GIULIA | | LIGURIA | | EMILIA-ROMAGNA | | TOSCANA | | UMBRIA | | MARCHE | | LAZIO | | ABRUZZO | | MOLISE | | CAMPANIA | | PUGLIA | | BASILICATA | | CALABRIA | | SICILIA | | SARDEGNA | | | |
| 1.3 | | None  Primary School | | --- | | Secondary School | | High School | | Degree | | | |
| 1.4 | | No /Housewife | | --- | | Yes | | | |
| **2.Health Status** | | | |
| 2.1 | Open ended question | | |
| 2.2 | Open ended question | | |
| 2.3 | Yes/No | | |
| 2.4 | Yes/No | | |
| 2.5 | Do you have: | | |
|  | a) Yes/No | | |
|  | b) Yes/No | | |
|  | c) Yes/No | | |
|  | d) Yes/No | | |
|  | e) Yes/No | | |
|  | f) Yes/No | | |
|  | h) Yes/No | | |
|  | e) Yes/No | | |
|  | If yes, what | | |
| **3.Family History** | | |  |
| 3.1 | Yes/No | | |
| 3.2 | Yes/No | | |
| **4. Infectious diseases** | | | |
| 4.1 | Yes/No/Do Not remember | | |
| 4.2 | Have you ever performed a test to verify whether you had or you have been vaccinated for: | | |
|  | a) Yes/No/Do Not remember | | |
|  | b) Yes/No/Do Not remember | | |
|  | f) Yes/No/Do Not remember | | |
| 4.3 | Did you get vaccination for: | | |
|  | a) Yes/No/Do Not remember | | |
|  | b) Yes/No/Do Not remember | | |
|  | c) Yes/No/Do Not remember | | |
|  | d) Yes/No/Do Not remember | | |
| **5. Lifestyle** | | | |
| 5.1 | Yes/No | | |
| 5.2 | Yes/No | | |
|  | | A beer or a glass of wine or a drink occasionally | | --- | | A beer or a glass of wine or an appetizer in the day | | More than a beer or a glass of wine or an appetizer in the day | | | |
| 5.3 | Yes/No | | |
| **6. Previous pregnancies** | | | |
|  | a) Yes/No | | |
|  | b) Yes/No | | |
|  | c) Yes/No | | |
| **Answer Questionnaire KNOWLEDGE** | | | |
| 1.1 | | Very informed  Informed  Poorly informed  Not at all informed | |
| 1.2 | | Be harmful to the fetus  Reduce the risk for birth defects  I do not know | |
| 1.3 | | Vitamin A and Fe  Folic Acid  Calcium | |
| 1.4 | | Before you get pregnant  As soon as you know you are pregnant  In the third month of pregnancy | |
| 1.5 | | Very informed  Informed  Poorly informed  Not at all informed | |
| 1.6 | | Before 18 years of age  After 40 years of age  I do not know | |
| 1.7 | | Very important  Important  Unimportant  I do not know | |
| 1.8 | | Low Blood Pressure  Diabetes During Pregnancy  Miscarriage and congenital malformations | |
| 1.9 | | Diabetes  High Blood Pressure  Both | |
| 1.10 | | None  Only reduced fertility, smoking does not reach the fetus  Reduced fertility, increased risk of spontaneous abortions and fetal malformations | |
| 1.11 | | All medications taken shortly before the pregnancy can cause birth defects in the child  All medications taken during the first months of pregnancy may cause birth defects in the child  Certain medications taken during the first months of pregnancy may cause birth defects the child  Some medications before pregnancy can cause birth defects in the child | |
| 1.12 | | Yes, if taken in large quantities (drinking alcohol every day)  Yes, even when taken in small quantities (eg. Glass of wine per week)  Alcohol is always harmful in pregnancy | |
| 1.13 | | Yes  No  Do not Know | |
| 1.14 | | | Chickenpox | | --- | | Rubella | | Both Chickenpox than Rubella | | |
